# Pathogenic gene variation spectrum and carrier screening for Wilson’s disease in Qingdao area

**DOI:** 10.1002/mgg3.1741

**Published:** 2021-07-09

**Authors:** Lingyan Qiao, Juan Ge, Cheng Li, Yusheng Liu, Conghui Hu, Sicui Hu, Wenjie Li, Tang Li

**Affiliations:** ^1^ Department of Pediatric Endocrinology and Genetic Metabolic Diseases Qingdao Women and Children’s Hospital Qingdao China; ^2^ Department of Pediatric Surgery The Affiliated Hospital of Qingdao University Qingdao China; ^3^ Neonatal Screening Laboratory Qingdao Women and Children’s Hospital Qingdao China

**Keywords:** ATP7B, carrier screening, genomic, Wilson's disease

## Abstract

**Background:**

Despite the increasing number of reports on the analysis of ATP7B variants, reports on carrier screening for Wilson's disease (WD, OMIM:277900) are rare.

**Methods:**

Peripheral blood samples were collected from 42 patients from Qingdao Women and Children's Hospital diagnosed with WD for direct sequencing of ATP7B gene. Twelve hotspot variants of ATP7B were selected for carrier screening in Qingdao area based on an analysis of information related to ATP7B variants and literature reports in China. We screened 5012 dried blood spots (DBSs) from asymptomatic newborns in Qingdao area to estimate carrier frequency. DNA was extracted from dried blood spots. Gene sequencing was performed using multiplex polymerase chain reaction (PCR) combined with second‐generation sequencing. The carrier frequency of each hotspot variant was calculated using the count data (expressed as number of carriers (%) obtained by direct counting.

**Results:**

The carrier frequency of 12 hotspot variants was 1.46% (95% CI: 1.16–1.83%). The ATP7B variant with the highest carrier frequency was c.2333G>T, accounting for 54.79% of all variants screened, followed by c.2975C>T and c.2621C>T, accounting for 17.81% and 15.07% of all variants screened, respectively.

**Conclusion:**

Carrier frequency of ATP7B gene pathogenic variants is high in the population in Qingdao area.

## Graphical abstract

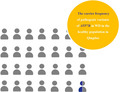

## 1 INTRODUCTION

Wilson's disease (WD, OMIM:277900) is an autosomal recessive disorder of copper metabolism caused by ATP7B gene (OMIM:606882) pathogenic mutations, which can lead to multiple organ and multisystem damage and seriously affect the quality of life of patients. WD is commonly seen in clinical practice in Qingdao area, but there are few reports on prevalence. As it is an autosomal recessive genetic disease, it is speculated that the genetic background of WD with a high prevalence in Qingdao area is probably related to the high carrier frequency of pathogenic variants of ATP7B gene in the population in this area. Despite the increasing number of reports on the analysis of ATP7B variants, reports on carrier screening for WD are rare. Therefore, in this study, the carrier frequency of hotspot variants of ATP7B in newborns with WD in Qingdao area from June 2016 to December 2018 was investigated and analyzed.

## 2 MATERIALS AND METHODS

### 2.1 Subjects

A total of 42 patients with WD diagnosed in the Department of Endocrinology and Metabolism, Qingdao Women and Children's Hospital, were enrolled in this study. WD was comprehensively diagnosed based on clinical manifestations, laboratory tests and genetic testing, according to the clinical guideline for WD proposed by Roberts and Schilsky (2008).

A cross‐sectional design was adopted. A total of 5020 newborns who were randomly selected by computer in Qingdao Women and Children's Hospital, the only Neonatal Screening Center in Qingdao area, from June 2016 to December 2018, were selected as the subjects in this study. After 8 were excluded, the remaining 5012 newborns were included in the carrier screening program. The exclusion criteria were as follows: (1) newborns with a family history of WD or liver diseases and (2) newborns whose mothers had a history of unexplained spontaneous abortion. The sample size was determined mainly according to the statistical formula “*n* = *t_α_* ^2^ *PQ*/*d* ^2^” (*α* = 0.05, *p* stands for estimated carrying rate, *Q* = 1 − *P*, *d* stands for allowable error, *d* = 0.003). All subjects gave their informed consent for inclusion before they participated in the study.

## 3 METHODS

### 3.1 Ethical compliance

The study was conducted in accordance with the Declaration of Helsinki, and the protocol was approved by the Ethics Committee of Qingdao Women and Children's Hospital (Project identification code: QFELL‐KY‐2018‐002).

### 3.2 Spectrum of pathogenic variants in WD and selection of hotspot variants for screening

A total of 42 patients from Qingdao Women and Children's Hospital diagnosed with WD were enrolled, mean age 7.4 ± 2.94 years old (18 males and 24 females). No enrolled patient had a family history of WD. Peripheral blood samples (2–3 ml each) were collected from patients and their parents and anticoagulated with ethylenediaminetetraacetic acid for direct sequencing of 21 exons in the coding region of the ATP7B gene (NM_000053.3). Hotspot variants of ATP7B were selected for carrier screening of WD in Qingdao area based on an analysis of information related to ATP7B variants, the calculation of allele frequency for each variant, and literature reports in China (Dong et al., 2016; Hua et al., 2016; Zong & Kong, 2016).

### 3.3 Carrier screening of pathogenic variants of ATP7B in Qingdao area

DNA extraction: Dried blood spots were generated from pricking the soles of newborns’ feet, and 3‐mm punches were sampled from these dried blood spots. DNA was extracted from the punches using a magnetic bead punch DNA extraction kit (CWBIO), and the DNA concentration and purity were measured using Nanoready (>10 mg/L and 1.7–2.1 (OD 260/280 ratio), respectively).

Gene sequencing: Gene sequencing was performed using multiplex polymerase chain reaction (PCR) combined with second‐generation sequencing; that is, specific loci were amplified in the first round of PCR, and biomarkers were added in the second round of PCR to construct libraries. DNA products were purified using a magnetic bead purification DNA kit (MyGenostics). After the establishment of the libraries, the capture efficiency was assessed with the Qubit concentration and agarose gel electrophoresis. KAPA library quantification kits (Beijing Pukairui Biotech Co., Ltd.) and KAPA SYBR Fast QPCR kits (Beijing Pukairui Biotech Co., Ltd.) for second‐generation sequencing were used to mix, dilute, and quantify the qualified libraries. Then, the enriched libraries were sequenced and analyzed using the Illumina novaS4 platform according to standard procedures.

### 3.4 Biological analysis

The raw images and raw data were converted to the original sequences through high‐throughput sequencing and base recognition; the sequences were then analyzed using cutadapt, BWA, Bamtools, Picard, GAT, and VarScan (mpileup2cns).

### 3.5 Statistical analysis

The carrier frequency of each hotspot variant was calculated using the count data (expressed as number of carriers (%)) obtained by direct counting; it was defined as the proportion of subjects with heterozygous mutations among all subjects and was approximately twice as high as the allele frequency. For the calculation of 95% confidence interval (CI) of the mutant carrier frequency, the online software was used (http://www.vassarstats.net/prop1.html).

## 4 RESULTS

### 4.1 Spectrum of pathogenic variants in WD and selection of hotspot variants for screening

**FIGURE 1:**
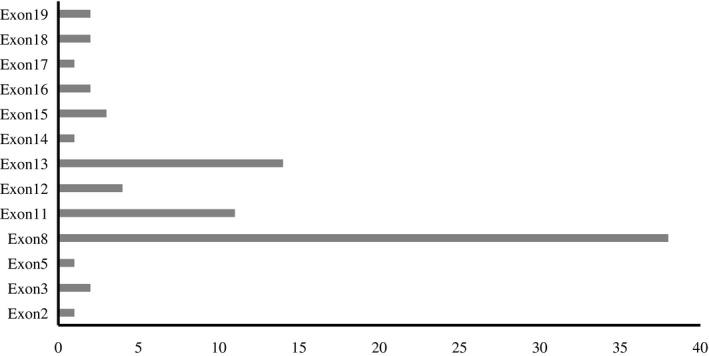
Mutation distribution within 21 exons of ATP7B

In 42 patients with WD, direct sequencing detected 28 ATP7B variants. The most common variant was c.2333G>T (p.R778L), accounting for 42.68% of all variants, followed by c.2975C>T (p.P992L), c.2621C>T (p.A874V), accounting for 14.63% and 8.54%, respectively. The variants were mostly detected in exons 8, 11, and 13, accounting for 46.34%, 13.41%, and 17.07%, respectively, totaling 76.82%. The distribution of ATP7B variants in the 21 exons is shown in Figure 1. The analysis results for the variants in the 42 patients with WD are provided in Table 1.

**TABLE 1:** Information of ATP7B mutations in 42 patients with Wilson's disease

Nucleotide change	Amino‐acid change	Location	Affected protein domain	Allele frequency (%)
c.2333G>T	p.R778L	Exon8	TM4	41.67 (35/84)
c.2975C>T	p.P992L	Exon13	TM6/Ph	14.29 (12/84)
c.2621C>T	p.A874V	Exon11	Td/TM5	8.33 (7/84)
c.3443T>C	p.I1148T	Exon16	ATP loop	2.38 (2/84)
c.3955C>T	p.R1319X	Exon19	ATP hinge/TM7	2.38 (2/84)
c.2755C>G	p.R919G	Exon12	Td/TM5	2.38 (2/84)
c.3316G>A	p.V1106I	Exon15	ATP loop	2.38 (2/84)
c.3029A>C	p.K1010T	Exon13	TM6/Ph	1.19 (1/84)
c.3809A>G	p.N1270S	Exon18	ATP hinge	1.19 (1/84)
c.2297C>T	p.T766M	Exon8	TM4	1.19 (1/84)
c.1745_1746delTA	p.I582Rfs*25	Exon5	Cu6	1.19 (1/84)
c.3247C>T	p.L1083F	Exon15	ATP loop	1.19 (1/84)
c.2730+1G>T	p.?	Exon11	Td/TM5	1.19 (1/84)
c.3767_3768insCA	p.Q1256Hfs	Exon18	ATP bind	1.19 (1/84)
c.2785A>G	p.I929V	Exon12	TM5	1.19 (1/84)
c.3044T>C	p.L1015P	Exon13	TM6/Ph	1.19 (1/84)
c.2299dupC	p.M769Hfs*26	Exon8	TM4	1.19 (1/84)
c.3089G>A	p.G1030D	Exon14	Ph	1.19 (1/84)
c.3646G>A	p.V1216M	Exon17	ATP bind	1.19 (1/84)
c.2662A>C	p.T888P	Exon11	Td/TM5	1.19 (1/84)
c.2310C>G	p.L770L	Exon8	TM4	1.19 (1/84)
c.1366G>C	p.V456L	Exon3	Cu4	1.19 (1/84)
c.1109_1110insACCTG	p.C370X	Exon2	Cu3/Cu4	1.19 (1/84)
c.1543+1G>T	p.?	Intron3	Cu5	1.19 (1/84)
c.2668G>A	p.V890M	Exon11	Td/TM5	1.19 (1/84)
c.2827G>A	p.G943S	Exon12	TM5	1.19 (1/84)
c.2730G>T	p.K910N	Exon11	Td/TM5	1.19 (1/84)

Abbreviations: Ch, ion channel; Cu, copper‐binding domain; Ph, phosphorylation loop; Td, transduction domain converting energy from ATP hydrolysis to cation transportation; TM, transmembrane domain.

The selected hotspot variants are all known pathogenic variants. Six variants detected in the patients in our center were selected, and another 6 variants (c.2804C>T, c.3884C>T, c.2924C>A, c.2930C>T, c.3532A>G, and c.994G>T) were selected based on their mention in the published literature (Dong et al., 2016; Hua et al., 2016; Zong & Kong, 2016). The selected variants for screening are shown in Table 2. The 12 selected mutations covered 70.24% of pathogenic variants in our center population and 47.63%–64.29% of pathogenic variants in patients reported in the literature (Table 2).

**TABLE 2:** Allele frequencies of 12 selected mutations in present and reported study

Nucleotide change	Amino‐acid change	Present study 84 alleles	Zong and Kong (2016) 70 alleles	Hua et al., (2016) 136 alleles	Dong et al., (2016) 1264 alleles
c.2333G>T	p.R778L	41.67%	45.70%	25.74%	29.67%
c.2621C>T	p.A874V	8.33%	7.10%	4.41%	3.56%
c.2975C>T	p.P992L	14.29%	7.10%	8.09%	14.56%
c.2804C>T	p.T935M	0	0	0.74%	7.12%
c.3443T>C	p.I1148T	2.38%	0	7.35%	3.32%
c.3884C>T	p.A1295V	0	0	0.74%	0.40%
c.3955C>T	p.R1319X	2.38%	1.40%	0	0.24%
c.3809A>G	p.N1270S	1.19%	0	2.94%	2.22%
c.2924C>A	p.S975Y	0	1.40%	0.74%	0.79%
c.2930C>T	p.T977M	0	1.40%	0	0.08%
c.3532A>G	p.T1178A	0	0	0	0.16%
c.994G>T	p.E332X	0	0	0	0.55%
Total		70.24%	64.29%	50.74%	47.63%

### 4.2 Carrier screening of pathogenic variants in WD

A total of 5012 newborns (2763 males and 2249 females) were screened for pathogenic variants, targeting 12 ATP7B variants. A total of 73 newborns (1.46%) carried pathogenic variants. Each patient carried only one variant, and none carried 2 or more hotspot variants. The carrier frequencies of 12 ATP7B variants are provided in Table 3. The c.2333G>T variant had the highest carrier frequency, accounting for 54.79% (40/73) of all targeted variants, followed by c.2975C>T and c.2621C>T, with carrier frequencies of 17.81% (13/73) and 15.07% (11/73), respectively. The three variants accounted for 87.67% (64/73) of all targeted variants.

**TABLE 3:** Carrier frequencies of 12 screening mutations in 5012 newborns

Nucleotide change	Amino‐acid change	No. of carriers	Carrier frequency (%)	95% CI (%)
c.2333G>T	p.R778L	40	0.80	0.59–1.09
c.2975C>T	p.P992L	13	0.26	0.15–0.44
c.2621C>T	p.A874V	11	0.22	0.12–0.39
c.2804C>T	p.T935M	3	0.06	0.02–0.18
c.3443T>C	p.I1148T	2	0.04	0.01–0.15
c.3532A>G	p.T1178A	2	0.04	0.01–0.15
c.3955C>T	p.R1319X	1	0.02	0–0.11
c.2924C>A	p.S975Y	1	0.02	0–0.11
c.3809A>G	p.N1270S	0	0	0
c.3884C>T	p.A1295V	0	0	0
c.2930C>T	p.T977 M	0	0	0
c.994G>T	p.E332X	0	0	0
Total		73	1.46	1.16–1.83

Abbreviations: CI, confidence interval.

## 5 DISCUSSION

WD is an inherited metabolic disease commonly seen in clinical practice. The metabolic disorder caused by ATP7B variants can result in multiple organ and multisystem damage, and delayed diagnosis and treatment of the disease results in poor patient prognosis. Although WD is one of the few genetic diseases that can be treated, there is currently no effective screening method.

Carrier screening is mainly conducted for autosomal recessive hereditary diseases. It is a primary preventive measure for reducing disease incidence by avoiding the birth of fetuses with high genetic risks through genetic counseling based on an understanding of genetic risks in a healthy population through biochemical methods and genetic testing. Carrier screening was initiated in the 1970s. With the progress of genetic testing technology, many genetic disorders can be screened using a single sample, regardless of race, and by 2016, the United States, Canada, Australia, and New Zealand all developed guidelines for expanded carrier screening (Edwards et al., 2015; Henneman et al., 2016; Wilson et al., 2016). Despite the long history of carrier screening in the world, information on carrier screening (except for thalassemia) is still scarce in China. The American College of Medical Genetics and Genomics (ACMG) and the American College of Obstetricians and Gynecologists (ACOG) guidelines recommend carrier screening for early‐onset severe diseases that cause cognitive impairment, require medical and surgical interventions, affect quality of life, and have an expected carrier frequency >1:100 (Committee on Genetics, 2017; Grody et al., 2013). WD possesses all these characteristics. This study is the first of carrier screening for WD in northern China.

When conducting carrier screening, there are two ways to detect a specific gene: whole‐genome screening and screening for hotspot variants of the causative gene. Current screening programs mainly focus on known hotspot variants of specific genes (Haque et al., 2016; Rose & Wick, 2018). There are numerous ATP7B mutations. More than 700 ATP7B mutations have been discovered, and most are point mutations (Fernando et al., 2020). However, the pathogenic variants for WD are concentrated in several hotspot variants, and the others are rare variants, with significant geographical differences (Ala, 2015). Research on ATP7B variants was carried out earlier in southern China than in northern China, with the subjects being mostly Han populations in southern China and with a major focus on exons 8, 12, 13, and 18. In contrast, there have been few relevant studies in northern China. Many studies in China have confirmed that the mutation p.R778L in exon 8 is a well‐recognized hotspot mutation, with a mutant allele frequency of 24~60% (Poujois & Woimant, 2018). In addition, the top three hotspot mutations are different in different regions, and the mutation rates for other exons are all low (Xie & Wu, 2017). The variants detected in our center were mostly in exons 8, 11, and 13, accounting for 46.34%, 13.41%, and 17.07%, respectively, and totaling 76.82% of all detected mutations, suggesting that this region might be a hotspot region for variants in the Qingdao area. Knowledge of the regional distribution of mutations of the Wilson's disease gene is important to design appropriate screening strategies (Ferenci, 2006). The spectrum of pathogenic variants in pediatric patients in Qingdao area was determined and analyzed in this study, and hotspot variants suitable for screening in this area were selected based on this spectrum and relevant Chinese literature to assess the coverage and accuracy of the detection methods through pre‐experiments. Multiplex PCR combined with second‐generation sequencing were used for carrier screening.

In this study, the carrier frequency of pathogenic variants of ATP7B was 1.46% (73/5012), that is, approximately 1/68 (95% CI: 1.16–1.83%), higher than the global carrier frequency of 1/90 (1.11%). In addition, Zhao et al. performed expanded screening for 11 recessive genetic diseases through whole‐genome sequencing of DNA derived from peripheral blood samples from 10476 healthy couples from five southern provinces in China (Zhao et al., 2019). In their results, the carrier frequency of WD was 1/51 (1.96%), higher than that obtained in this study. However, the carrier frequency of WD in this study was higher than the overall carrier frequencies of 1/85 (95% CI: 0.71–1.65%) and 1.15% (95% CI: 1.03–1.28%) in Korea, as estimated by Song et al. and Jang et al. based on the screening of hotspot variants (Jang et al., 2017; Song et al., 2012). Among the nine high‐risk serious genetic diseases that the ACOG and ACMG recommend for screening in Jews, the carrier frequency of Gaucher disease was 1/15, the carrier frequencies of Tay‐Sachs disease, cystic fibrosis and two other genetic diseases ranged from 1/40 to 1/30, and the carrier frequencies for another four diseases were each approximately 1/100 (ACOG Committee on Genetics, 2009). The above results indicate that compared with other studies of carrier screening, the carrier frequency of ATP7B gene pathogenic variants is high in the population in Qingdao area. However, does a high carrier frequency of pathogenic variants lead to a high clinical prevalence? The answer is very likely “no.” The results reported by Sandahl et al. showed that the internationally recognized prevalence rate of 1/30,000 is still valid in Asia (Sandahl et al., 2020). In some population‐based studies, the prevalence of genetic diseases is 3–4 times the clinical estimate, suggesting that penetrance requires further evaluation. Wallace et al. found that after ATP7B variants with low penetrance were excluded, the revised prevalence rate was approximately 1/20,000, that the prevalence rate of WD was higher among Asians and Ashkenazi Jews, and that the prevalence rates of WD in the UK and France were lower than the reported values (Coffey et al., 2013; Collet et al., 2018). The results indicated that the difference in the penetrance of variants may explain the differences between the genetic prevalence and actual prevalence of WD (Wallace & Dooley, 2020). Therefore, the hotspot variants selected in this study are all known pathogenic variants, further confirming that the relatively common clinical genetic background of WD in Qingdao area is likely to be the basis of the high carrier frequency of pathogenic variants in the population in this area.

The ATP7B variant with the highest carrier frequency was c.2333G>T, accounting for 54.79% of all variants screened, followed by c.2975C>T and c.2621C>T, accounting for 17.81% and 15.07% of all variants screened, respectively. The above three hotspot variants accounted for 87.67% of all variants screened, a percentage that is consistent with that for the top three most common genetic variants detected in WD patients in our center. In addition, four ATP7B variants were not detected in any subject, a result that may be caused by two factors. First, genetic variants are numerous and sporadic, and there are significant regional differences in hotspot variants. Due to the lack of a large‐sample‐based variant spectrum specific for the Qingdao area, the variants for screening could only be selected based on the variants detected in our center and variants reported in the Chinese literature. Three (c.3884C>T, c.2930C>T, and c.994G>T) out of the four ATP7B variants that were not detected in any subject were all selected based on the literature, suggesting that they may not be hotspot variants in Qingdao area. The inclusion of these variants in screening will result in a lower detection rate for the targeted gene. Second, the c.3809A>G variant, which was selected based on common findings from our research center and literature reports, was also not detected in any subject, a result that may be due to the limited number of samples, making it difficult to detect some variants.

This study aimed to understand the carrier frequency of pathogenic variants of ATP7B in WD in the healthy population in Qingdao area, to elucidate the causes for the high incidence of WD in this region and to provide preliminary experience for the implementation of carrier screening for WD. Screening of carriers in a normal population is of great significance, that is, understanding the status of causative genes in normal people and assessing the risk of progeny suffering from genetic diseases can provide information for genetic counseling and for families regarding future fertility decisions. When parents are both carriers of a serious genetic disease, routine prenatal diagnosis during early pregnancy is recommended, and preimplantation genetic diagnosis should be performed if necessary to prevent the transmission of pathogenic variants to the next generation and the birth of fetuses with this genetic disease (Nazareth et al., 2015). Therefore, carrier screening is classified as primary prevention and is a fundamental measure for the prevention and control of inherited metabolic diseases. However, in China, great challenges still exist regarding various aspects of the universal promotion of carrier screening, such as public awareness and education, informed consent, interpretation of results, and genetic counseling.

## CONFLICT OF INTEREST

The authors declare that they have no conflicts of interest.

## AUTHOR CONTRIBUTIONS

All authors contributed to the study conception and design. Material preparation was performed by [Guan Ge] and [Cheng Li]. Data collection was performed by [Lingyan Qiao] and analysis was performed by [Yusheng Liu] [Conghui Hu] [Sicui Hu] and [Wenjie Li]. The first draft of the manuscript was written by [Lingyan Qiao] and [Tang Li]. All authors commented on previous versions of the manuscript. All authors read and approved the final manuscript.

## ETHICS STATEMENT

The study was conducted in accordance with the Declaration of Helsinki, and the protocol was approved by the Ethics Committee of Qingdao Women and Children's Hospital. Written informed consent was provided by the participant.

## Data Availability

The corresponding author had full access to all the data in the study and takes responsibility for the integrity of the data and the accuracy of the data analysis.
